# On the Effect of Chemical Composition on the Desorption of Superabsorbent Hydrogels in Contact with a Porous Cementitious Material

**DOI:** 10.3390/gels4030070

**Published:** 2018-08-17

**Authors:** Khashayar Farzanian, Ali Ghahremaninezhad

**Affiliations:** Department of Civil, Architectural and Environmental Engineering, University of Miami, Coral Gables, FL 33146, USA; kxf148@miami.edu

**Keywords:** hydrogels, capillary forces, desorption, cementitious materials

## Abstract

The behavior of poly(sodium acrylate-co-acrylamide) copolymer hydrogels with varied chemical compositions in artificial pore solutions with three different pH values is examined. The absorption, chemical characteristics, mechanical stiffness, and desorption of the hydrogels in contact with a porous cementitious material were investigated. It was observed that the surface characteristics of the hydrogels play an important role in the desorption of hydrogels due to the capillary forces. It was shown that in the hydrogel systems studied here, the bonding between the hydrogels and the porous cementitious material is improved with an increase in the content of acrylamide in the hydrogels, and this results in an increased desorption rate of the hydrogels.

## 1. Introduction

Superabsorbent polymers (SAP) have gained significant interest as an internal curing agent in the past several years due to their capability to mitigate autogenous shrinkage in cementitious materials with low water to cement ratios [1,2,3,4,5,6,7,8]. Prior investigations found that the use of SAP can potentially improve the hydration of cementitious materials [3,9]. The effect of SAP on the transport properties of cementitious materials was studied by the researchers [9,10,11]. The potential of SAP for self-sealing [12,13,14] and self-healing [15,16] applications have also been investigated by researchers. SAP is considered a subcategory of hydrogels, which consist of hydrophilic polymeric networks that possess a high capacity of water absorption up to 1500 times of their dry mass [5,17,18,19]. 

The SAP used in cementitious materials are primarily based on poly(acrylate-co-acrylamide) copolymer hydrogels [2,17,20,21]. This type of hydrogels is polyelectrolyte and their behavior is strongly dependent on the pH and ionic compositions of the environment [5,7,17,18,21,22]. When these hydrogels come into contact with distilled water, the polymer networks of the hydrogels become deprotonated and gain negative charges, which result in repulsive forces within the polymer networks. The repulsive forces within the networks promote water absorption into the hydrogels [17]. When cations exist in the solution, they screen the repulsive electrostatic forces within the polymer networks; in addition, some divalents and trivalents have been shown to form complexes with the anionic groups of the polymer networks [7,19,23]. Thus, the screening effect and complexation of cations cause a reduction in the absorption capacity of the hydrogels [5,17,18,19].

The thermodynamics and kinetics dictating the behavior of hydrogels is influenced by the chemical composition of the polymeric networks. Tuning the chemical composition of the hydrogels provides a reliable means to adjust the absorption capacity and kinetics of hydrogels according to the chemistry of the environment. Polymer networks stretching, mixing of the networks and solvent, and the ionic interactions are the factors determining the free energy of hydrogels [24].

The absorption capacity and rate of hydrogels play a critical role in determining the properties of cementitious materials. The mechanical strength of cementitious materials can be adversely affected when hydrogels with a large water retention ability, which create large macrovoids in the microstructure, are used [5,10,25,26,27,28]. The rate of desorption of water from hydrogels during hydration is an important factor for proper internal curing and mitigation of autogenous shrinkage in cementitious materials [5,7,29]. Thus, a comprehensive understanding of the behavior of hydrogels taking into account the chemistry of cementitious materials is of utmost importance to ensure the proper design of hydrogels to achieve desired characteristics. 

In the early stage of hydration and before solid skeleton development in the microstructure of cementitious materials, the behavior of hydrogels is primarily determined by the chemistry of the pore solution, interaction with cement particles and mechanical constraint imparted by the fresh cementitious matrix [5,7,17,18,19,21,30,31]. After setting and start of solid skeleton development, self-desiccation results in creation of capillary forces in the microstructure. At this stage, the desorption of hydrogels is primarily governed by the capillary forces. 

Several previous studies investigated the link between hydrogel chemical compositions, chemistry of the solution, and the absorption characteristics of hydrogels [5,7,17,18,19,21,30]. However, the desorption behavior of hydrogels has not received adequate attention in the literature. A few recent investigations examined the desorption of hydrogels in pore solutions or in air with controlled relative humidity [32,33]. Neutron tomography [34] and neutron radiography [35] were also used to image the desorption of hydrogels in hydrating cementitious materials. Using Nuclear Magnetic Resonance (NMR), the water release from SAP towards cementitious matrix during the hydration reaction was studied [29,36]. However, the underlying mechanisms affecting the desorption of hydrogels due to the capillary forces have scarcely been examined in the past [31,37]. 

This paper examines the desorption of hydrogels in contact with a porous cementitious material and its relationship with the chemical composition of the hydrogels. Hydrogels with three different chemical compositions were synthesized and used in the experiments. The effect of pH of a synthetic pore solution on the absorption, mechanical stiffness, chemical characteristics, and desorption of the hydrogels was investigated. The influence of the capillary action on hydrogel desorption was compared to hydrogel desorption without the capillary effect.

## 2. Results and Discussion

### 2.1. Hydrogel Absorption in Synthetic Pore Solutions

**Figure 1:**
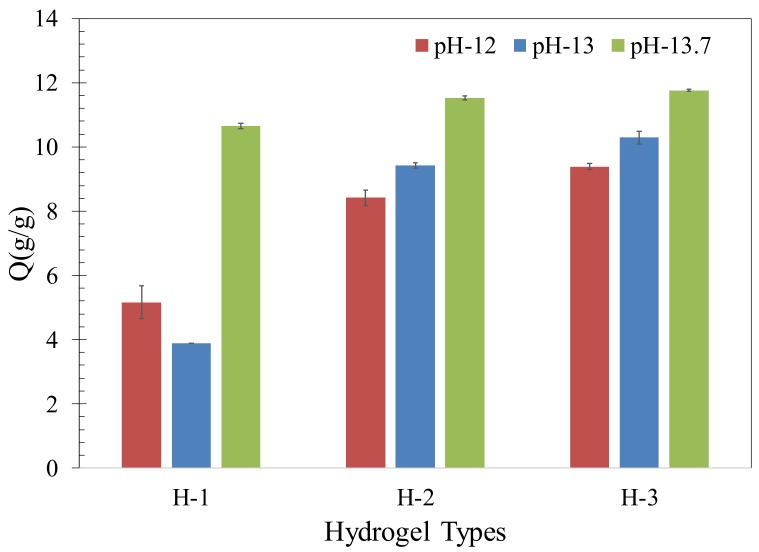
Absorption of the different hydrogels in synthetic pore solutions with varied pH values.

**Figure 2:**
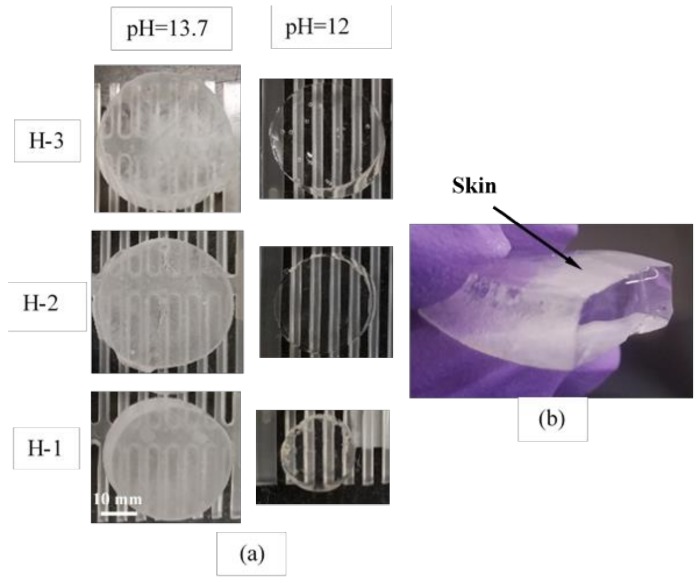
(**a**) Images of the hydrogel disks after absorption in synthetic pore solutions with pH = 12 and pH = 13.7. (**b**) Image showing the formation of a skin on the surface of a hydrogel disk swollen in the synthetic pore solution with pH = 13.7.

The absorption results of the hydrogels with different compositions swollen in synthetic pore solutions with pH of 12, 13, and 13.7 are shown in Figure 1. The absorption of H-1, H-2, and H-3 in distilled water is 398, 313, and 165 (g/g), respectively. The images of the swollen hydrogels are shown in Figure 2. The formation of a thin skin on the surface of the hydrogels at pH = 13.7 can be seen from the images. More discussion regarding this skin and its influence on the desorption of the hydrogels will be provided later in the paper. It is seen that the absorption of hydrogels in distilled water increases with an increase in the concentration of acrylic acid (AA). However, the absorption of the hydrogels decreases with increasing concentration of AA in the synthetic pore solutions at all pH values studied here. It is noted that H-3 and H-2 showed an increase in the final absorption with increasing pH from 12 to 13.7. However, a different behavior is observed in the absorption of H-1. At pH of 13, H-1 exhibited a small decrease in absorption compared to that at pH of 12, but then showed an increase at pH of 13.7. 

At high pH values (more than 12), the amide groups of acrylamide (AM) hydrolyze to carboxylic acid groups; thus, an increase in the anionic carboxylic groups promotes the electrostatic repulsion within the polymer networks resulting in an increase in the water absorption of the hydrogels. In addition, at high pH values studied here, the amide groups of the primary crosslinks can hydrolyze resulting in the breakage of the primary crosslinks and a reduction in the elastic stiffness of the polymer networks [33]. As a result of a reduction in elastic stiffness, more water can be absorbed within the polymer networks. However, it should be noted that with increasing reduction of stiffness, there may be a limit beyond which the structural integrity of the polymer networks is significantly reduced and the polymer networks are no longer able to retain water; in that case, a reduction in the absorption of the hydrogels is expected. In the presence of ions, such as Ca^2+^, Na^+^, and K^+^ in the solution, the absorption is reduced due to the screening effect of the positively charged ions as well as complexation between anionic groups and primarily divalent ion Ca^2+^ [38,39,40]. It has been suggested that the presence of divalent ions reduces the hydrolysis of primary crosslinks at high pH [33] and this could reduce the influence of hydrolysis on the hydrogel absorption.

It appears that in H-2 and H-3 with a high concentration of AM monomers, the absorption promoting effects outweighed that of the absorption reducing effects and the increase in pH from 12 to 13 and to 13.7 resulted in a continuous increase in the absorption of these hydrogels. However, in H-1 with a high concentration of AA monomers, the competition between the above interactions favored a slight reduction in absorption at pH of 13 compared to that at pH of 12. Since almost all carboxylic acid groups of AA monomers are expected to be deprotonated at pH values greater than 5, carboxylic acid groups are not expected to influence the absorption in the pH range studied here. Thus, the absorption behavior of H-1 is primarily affected by the competition between the hydrolysis of the primary crosslinks and ionic crosslink formation, as a result of complexation.

It should be noted that the kinetics associated with these mechanisms is dependent on the size of hydrogels, and as a result, the size of hydrogels is anticipated to affect the time dependent behavior of the hydrogels. The discussion provided above relates to the equilibrium state of the hydrogels after 48 h. 

### 2.2. Mechanical Behavior

**Figure 3:**
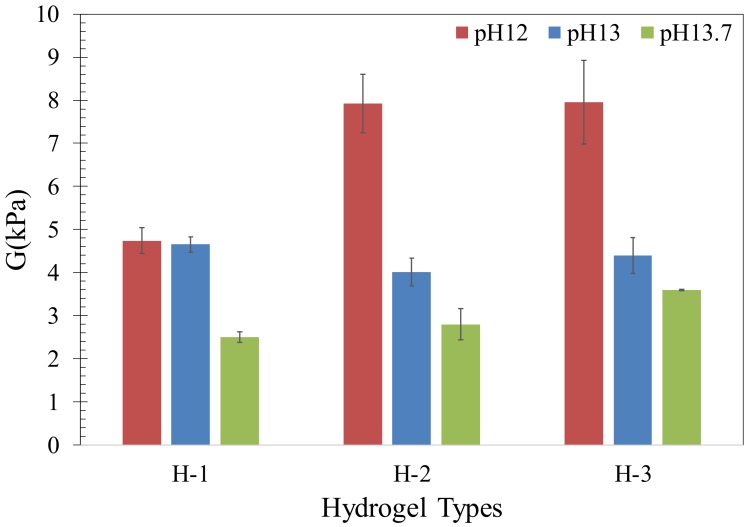
Shear modulus of hydrogels swollen in the synthetic pore solutions.

The shear modulus of the hydrogels after absorption in the synthetic pore solutions is shown in Figure 3. It is observed that all hydrogels maintained their overall mechanical integrity and did not show any indication of dissolution, at least at the macroscale, in the solutions examined in this study. A decrease in shear modulus with increasing pH in H-2 and H-3 can be noted; however, in H-1, a different behavior is realized. The shear modulus of H-1 was similar at pH = 12 and pH = 13, and then decreased at pH = 13.7. 

The mechanical response of hydrogels is affected by the primary covalent crosslink density and the ionic crosslink density of the polymeric networks [41,42,43]. Thus, the mechanical response of hydrogels is intimately dependent on the water absorption of hydrogels as the number of crosslinks per unit volume of hydrogels decreases with increasing water content [17,44]. It is seen that there is a close correlation between the shear modulus and the absorption of each hydrogel in different solutions. 

It is interesting to note the concurrent increase in absorption and shear modulus in the hydrogels with increasing concentration of AA at pH of 12. In general, shear modulus is inversely related to absorption [17,44]; thus the observed increase in shear modulus at pH of 12 is unlikely to be attributed to the density of crosslinks given the same composition of the primary crosslinking agent (*N*,*N*′-methylenebisacrylamide, MBA) was used in the synthesis of the hydrogels. It is probable that the structural characteristics of the polymer networks are responsible for the observed trend in the shear modulus of the hydrogels at pH of 12.

### 2.3. FTIR Analysis

**Figure 4:**
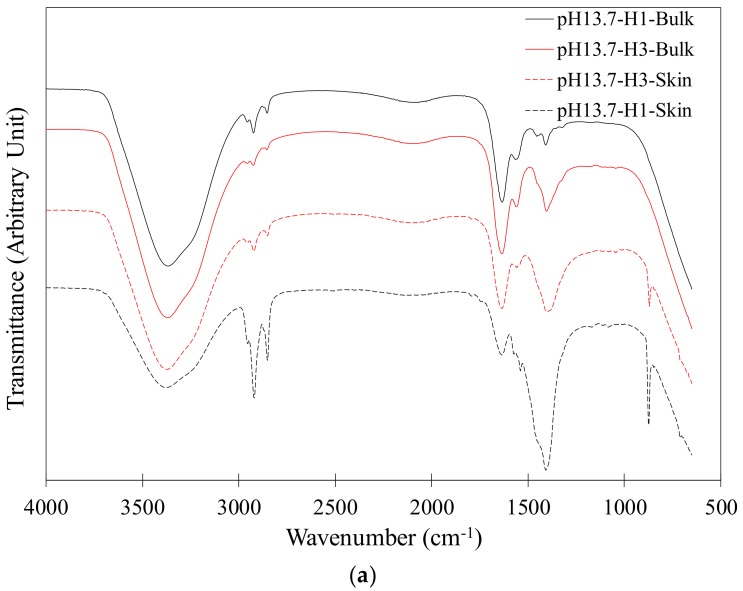

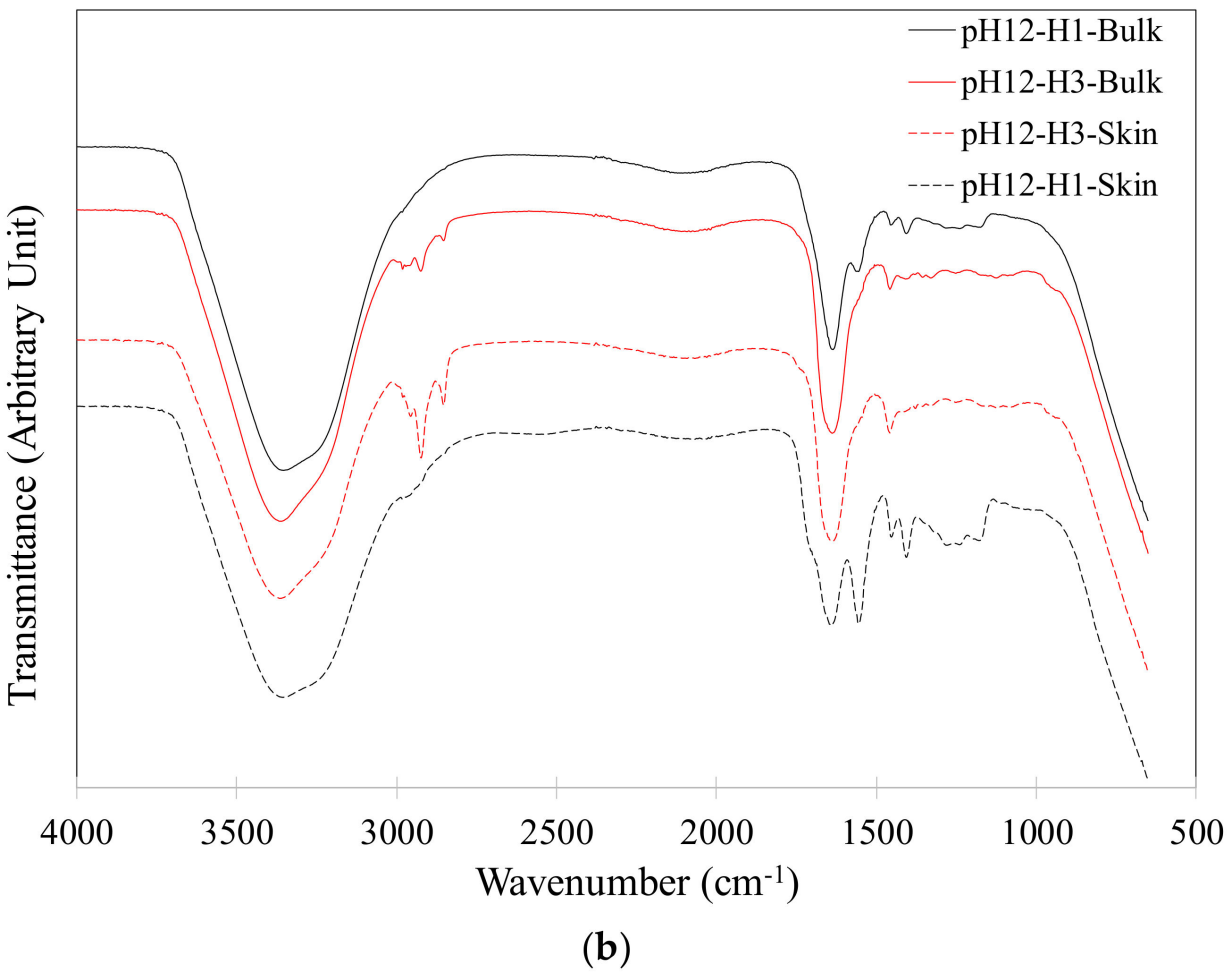
FTIR spectra of the skin and interior bulk of H-1 and H-3 after absorption in synthetic pore solutions with (**a**) pH = 13.7 and (**b**) pH = 12.

The FTIR spectra of H-1 and H-3 swollen in the synthetic pore solutions with a pH of 13.7 and 12 are shown in Figure 4a,b. The spectra of the skin and the bulk are included in the figures. It can be seen from Figure 4a that general features are observed in the spectra of the interior bulk of H-1 and H-3 [45]. A broad peak between 3000 and 3600 cm^−1^ is observed in all spectra; this peak is attributed to the O–H stretching indicative of hydrogen bonds in the hydrogel molecular structure due to high water content. Bands at about 1400 and 1560 cm^−1^ correspond to carboxylate group stretching [18,33,46]. At 1635–1645 cm^−1^, a stretching of the C=O group from the acrylamide unit appears in all spectra [47]. An interesting observation is made of a peak at about 873 cm^−1^ appearing only in the skin spectra, as shown in Figure 4a. This peak is attributed to calcium carbonate (CaCO_3_) [48] and appears stronger in H-1 than in H-3. The formation of CaCO_3_ provides evidence for the complexation between Ca^2+^ and the anionic carboxylic groups on the hydrogel polymer networks. The occurrence of carbonation during swelling of SAP hydrogels in a cement filtrate was also observed in a previous study [49]. The appearance of the CaCO_3_ peak only on the surface of the hydrogels could be due to a higher density of Ca^2+^ complexes on the surface compared to the bulk or a higher concentration of carbonate ions on the surface; the latter seems to be a more plausible reason for this observation as the CO_2_ diffusion occurs from the surface. The stronger peak of CaCO_3_ in H-1 compared to H-3 can be attributed to a higher density of complexes in H-1 than H-3, which is to be expected due to a higher concentration of AA monomers in the composition of H-1 than H-3. 

The spectra of the skin of H-1 and H-3 at pH of 12 are also shown in Figure 4b. No significant difference was observed between the skin and bulk spectra in H-1 or H-3 at pH of 12. It is worth noting the absence of the CaCO_3_ band in the skin spectra of H-1 and H-3. An explanation for this could be increased Ca^2+^ complexation in the hydrogels at pH of 13.7 compared to pH of 12.

### 2.4. Desorption with Contact with Cement Paste Blocks

**Figure 5:**
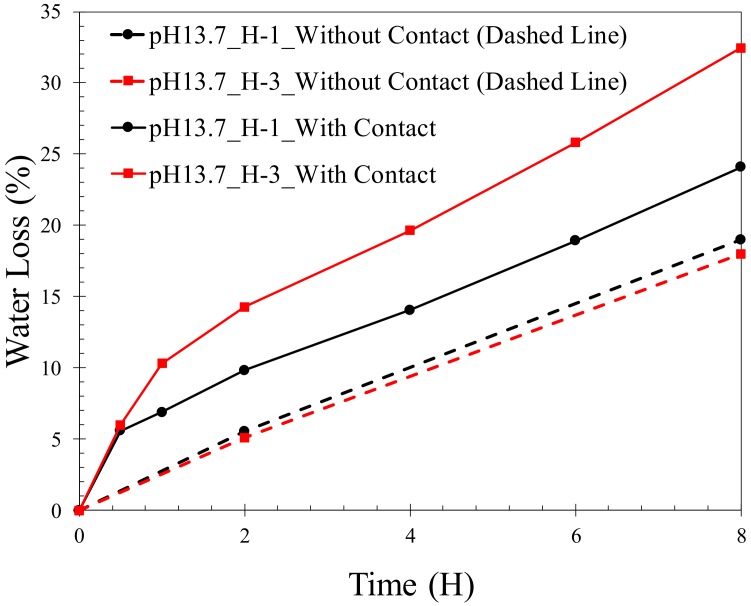
Desorption of H-1 and H-3 hydrogel disks with contact and without contact (control) with a cement paste block.

The desorption of hydrogels, H-1 and H-3, swollen at pH of 13.7 is depicted in Figure 5. The results shown in this figure correspond to the hydrogels with contact with the blocks (sandwiched) and without contact (in air). It is noted that contact with the cement paste blocks increased the desorption rate of the hydrogels compared to the case without contact. The difference in the rate of desorption of hydrogels as a result of contact with the blocks is more pronounced in H-3 compared to H-1 in the first 8 h. The enhanced rate of desorption is attributed to the effect of the capillary forces at the interface between the hydrogels and the porous blocks. As a result of the capillary suction, a thin layer of the soft hydrogel adjacent to the interface is pulled against the hard surface of the block squeezing the water out of the thin layer [37,50]. This mechanism is thought to be responsible for the faster release of water from the hydrogels when they are in contact with the blocks compared to when they are not. 

It is interesting to note that while the desorption rate of the two hydrogels when there is no contact with the blocks is close to each other, as seen from Figure 5, a difference exists in the desorption rate when there is contact. When there is no contact with the blocks, the similar water loss behavior of the two hydrogels points to their similar bulk diffusion rate, considering the small difference in the initial water content at the beginning of desorption of the two hydrogels.

A potential explanation for the observed behavior of the hydrogels when there is contact with the blocks could be offered by examining the characteristics of the hydrogel surface swollen in pH of 13.7. As discussed previously, visual observations as well as the FTIR analysis of the hydrogel surface suggested a more pronounced formation of a skin, potentially made of a Ca^2+^ rich compound on the surface of H-1 rather than H-3. It is expected that the relatively hard thin skin of Ca^2+^ compound could affect the contact surface between the hydrogel and the capillary pores on the surface of the cement paste blocks. Previous investigations related to the contact adhesion due to the capillary forces at the interface between an elastomer and a hard solid with surface asperities showed a decrease in adhesion forces when the elastic stiffness of the elastomer was increased [51]. A soft elastomer can be pulled into a closer contact with the hard solid surface; therefore, with decreasing elastic stiffness, the contact area is increased between the soft solid and the hard surface. Therefore, it is speculated that the capillary effect on the surface of H-1 is expected to be reduced compared to that on the surface of H-3 due to a more pronounced skin formation on H-1. It seems logical that the more pronounced formation of the skin on the surface of H-1 than H-3 is responsible for the slower desorption of H-1 than H-3.

### 2.5. Desorption Test with a Fixed Gap

**Figure 6:**
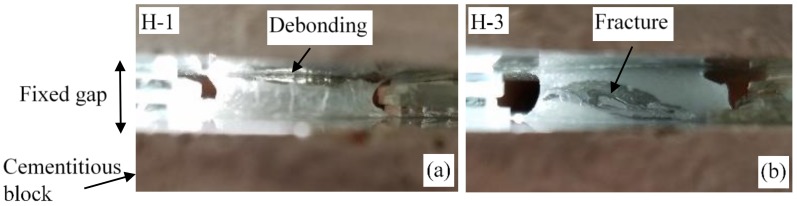
Images showing (**a**) debonding between H-1 and the cement paste block and (**b**) a fracture in H-3 in the desorption experiment setup with a fixed gap between the top and bottom cement paste blocks.

In this section, the desorption of H-1 and H-3 sandwiched between cement paste blocks with a fixed gap is discussed. As mentioned previously, this experiment was performed to broadly mimic the desorption condition of hydrogels enclosed by a constant sized macrovoid. The images depicting the desorption of H-1 and H-3 swollen at pH of 13.7 are presented in Figure 6. At pH of 13.7, debonding over a large portion of the interface between H-1 and the top block occurred; on the other hand, a fracture is seen inside H-3. The weak adhesion due to a more pronounced hard surface skin formed on H-1 rather than H-3, as discussed previously, could be the reason for the interface debonding in H-1. The debonding results in a reduction in the contact surface between the hydrogel and the cement paste block and as a consequence, the capillary suction on the hydrogel is decreased. On the other hand, the desorption of H-3 revealed a contrasting mechanism; H-3 remained bonded to the blocks; in this case, volume reduction as a result of water loss caused tensile stresses within H-3. With continued desorption, at a critical tensile stress, a fracture was nucleated and developed inside H-3.

The distinct mechanisms observed in the desorption behavior of H-2 and H-3 play an important role in their desorption. The strong bonding between H-3 and the blocks is expected to have improved the rate of desorption compared to H-2, which experienced debonding. Additionally, the effective diffusion length is reduced when a fracture occurs and the hydrogel is broken into smaller parts. 

It should be noted that the processes related to debonding and fracture formation in the hydrogels are dependent on the size and geometry of hydrogels and the surrounding macrovoids; however, the focus of the experiments was to highlight the basic effect of debonding and fracture formation on the desorption behavior of hydrogels. It is realized that the mechanical response, such as stiffness and fracture toughness, of hydrogels could play an important role in the desorption of hydrogels in a cementitious matrix. Further investigations are necessary to elucidate the individual and combined effect of other relevant factors on the desorption of hydrogels in cementitious materials.

It is worth noting that the physical characteristics and chemical composition of hydration products could greatly influence the interaction between hydrogels and the cementitious matrix. For example, in a previous study [4], the growth and intrusion of hydration phases including calcium hydroxide and calcium carbonate towards the SAP macrovoids has been noted. Nonetheless, the primary focus of the current paper was to provide insights into the underlying mechanisms influencing the desorption behavior of hydrogels interacting with an unsaturated porous solid. Such insights will increase our knowledge of hydrogel desorption in cement-based material systems.

## 3. Conclusions

The effect of chemical composition of hydrogels on their behavior in artificial pore solutions with varied pH and their desorption in contact with a porous cementitious material were studied. It was shown that hydrogels with a higher concentration of AA showed a lower desorption rate when in contact with a porous cementitious material. A potential reason for this observation was related to the effect of the pore solution on the surface characteristics of the hydrogels. A skin was observed to form on the surface of hydrogels swollen in high pH pore solutions and this skin was more pronounced in hydrogels with a higher concentration of AA. The presence of this skin at the interface between the hydrogels and the cementitious matrix influenced the contact at the interface, and as a result, the effect of capillary forces on the hydrogel desorption. The interface bonding with the cementitious matrix is expected to influence the desorption rate of hydrogels. The results from this study help elucidate the fundamental processes affecting the desorption behavior of hydrogels in cementitious materials.

## 4. Experiments

### 4.1. Materials

#### 4.1.1. Hydrogels

The chemicals used in the synthesis of the hydrogels were purchased from Sigma–Aldrich (St. Louis, MO, USA) and used as received. Poly(sodium acrylate-co-acrylamide) copolymers with three different chemical compositions were used in this study and their compositions are listed in Table 1. Hydrogels were synthesized using the free radical polymerization method, as described in Horkay et al. [52]. Main monomers were AA and AM. Hydrogels with three different AA/AM ratios were synthesized. A solution of AA in 50 mL distilled water was first partially neutralized with a 13.5% sodium hydroxide (NaOH) solution. Then, AM and 0.025 g of *N*,*N*′-methylenebisacrylamide (MBA), which served as the crosslinking agent, were added to the solution, stirred for 30 min, and degassed with argon for 3–5 min. In order to initiate the polymerization, 0.064 g of ammonium persulfate was added to the solution, stirred for 5 min, and then poured in thin layers using specific glass molds. The solutions were gelated in an oven at 60 °C for 3 h. After gelation, the surface of the hydrogel layers was cleaned using alcohol to remove residual/unreacted monomers. In order to remove wrinkles or creases from the hydrogel surface, the hydrogels were soaked in distilled water for 1–3 h, and then placed on a plastic mesh. The next day, the hydrogels were surface cleaned with alcohol and punched into 16 mm diameter disks. Hydrogel disks with varied thicknesses were prepared and used in different experiments. All disks were dried overnight on a plastic mesh in an oven at 60 °C. The plastic mesh was treated with silicone oil to prevent any adhesion with the hydrogel disks and to allow for uniform drying of the hydrogel disks to achieve non-deformed dried hydrogel disks. All hydrogel disks had the same initial dry dimensions of 9 mm in diameter. Hydrogel disks with a thickness of 1.8 mm in the dry condition were prepared to be used in the absorption, FTIR and shear modulus measurements, and thin hydrogel disks with a 0.25–0.28 mm dry thickness were prepared to be used in the capillary desorption experiment, as described in section 2.4. Thin hydrogel disks were used in the capillary desorption experiment so that their thickness was comparable to the SAP particle size used in the internal curing applications.

#### 4.1.2. Cement Paste Blocks

Cement paste blocks were prepared in order to examine the desorption of hydrogels due to the capillary effect. 50 mm cement paste cubes with a water/cement ratio of 0.45 and addition of a lignosulfonate-based superplasticizer at a concentration of 0.5%, by cement mass, were prepared. At the age of 7 days, the cement paste cubes were soaked in acetone for 12 h to stop hydration, and then saw cut into three blocks. The surface of the blocks was polished using sand papers of 80, 180, 320, 600, 1200 grit sizes. After ultrasonication in ethanol for 20 min and drying in an oven at 40 °C for two days, the blocks were placed in a drybox with a relative humidity of 75% for more than 30 days. A saturated solution bath of sodium chloride was used to maintain the relative humidity of the drybox.

#### 4.1.3. Synthetic Pore Solutions with Varied pH

In order to examine the effect of pH on the behavior of hydrogels, synthetic pore solutions with varied pH levels of 12, 13, and 13.7 were prepared using Ca(OH)_2_, NaCl, and KCl. All solutions were saturated with Ca(OH)_2_ to simulate conditions in ordinary Portland cement concrete [53]. The synthetic pore solutions were prepared with concentrations of [Na^+^] = 400 mM and [K^+^] = 400 mM. Ca(OH)_2_ was oversaturated at 2.24 g/L as suggested by [54]. The pH of the solution was adjusted using NaOH and HCL solutions. The absorption of hydrogels in the synthetic pore solutions with varied pH levels was measured after about 48 h in solutions to allow samples to reach equilibrium. The solutions were initially purged and kept sealed during the experiments. The absorption was measured using the following equation:

(1) $$Q=\frac{\left(m_{saturated}-m_{dry}\right)}{m_{dry}}$$

where *m_saturated_* is the mass of swollen hydrogel and *m_dry_* is the mass of the dry hydrogel. The average of two replicates was calculated and reported.

### 4.2. Mechanical Measurement

The compressive tests were utilized to measure the mechanical stiffness of the hydrogels after swelling in varied artificial pore solutions. Fully swollen hydrogels were subjected to uniaxial compression under a displacement controlled condition at a rate of 0.0195 mm/s. Silicone oil was applied to the interface between the hydrogel surfaces and the loading plates to remove any friction. The shear modulus (*G*) of the hydrogels was calculated using the force-displacement measurements assuming a rubber elasticity constitutive behavior as follows [52,55]:

(2) $$\sigma =G\left(\lambda -\lambda^{-2}\right)$$

where *σ* is nominal stress and *λ* is the ratio of deformed thickness to undeformed thickness. Two replicates were used in the compressive test and the average value was reported.

### 4.3. FTIR Analysis

In order to elucidate the chemical effect of the pore solutions on the hydrogels, FTIR was employed. Both the surface and the interior bulk of the hydrogels were subjected to the FTIR analysis using a PerkinElmer Paragon 1000 FTIR with the ATR accessory. This is motivated by the changes observed on the surface of hydrogels in contact with alkaline solutions [17,31,56]. The surface of the hydrogels was placed directly on the instrument; in order to scan the interior of the hydrogels, the hydrogel disks were notched from the edge using a blade and the top layer was peeled off to obtain a smooth surface.

### 4.4. Desorption of Hydrogels with Contact with Cement Paste Blocks

**Figure 7:**
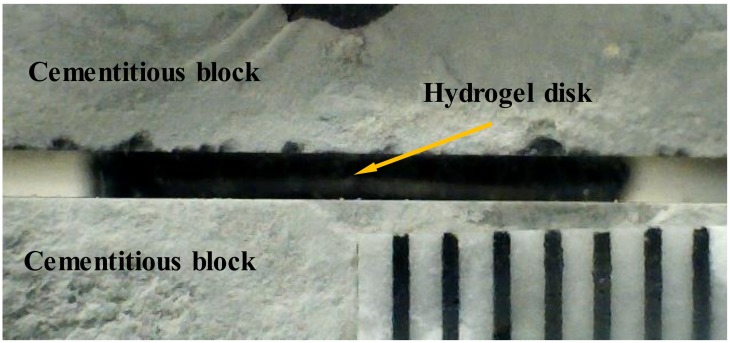
Image of the setup used in the desorption experiments with contact with cement paste blocks.

In order to gain insights into the effect of the composition of hydrogels on desorption when in contact with cementitious materials, hydrogels at the swollen state were removed from the synthetic pore solutions and sandwiched between two cement paste blocks. The sandwiched hydrogels were then stored in a drybox with a relative humidity of 75%. The change in the distance between the top and bottom blocks was used to determine the change in the thickness of the hydrogels, which can be used to calculate desorption. The images of the sandwiched hydrogels were taken with a camera with a resolution of 640 × 480 pixels. Figure 7 depicts the setup used in the desorption experiments. The water loss of the sandwiched hydrogels was measured as (1 − *H_i_*/*H*_0_) × 100 where *Hi* and *H*_0_ are the height of hydrogel at different times and at start of the desorption, respectively. It should be noted that the volumetric change of sandwiched hydrogels occurred primarily in the thickness direction; however, the volumetric changes in the planar dimensions could also take place but this was not able to be measured using the setup adopted in the experiments. Therefore, the actual desorption of the sandwiched hydrogels is slightly higher than that estimated based on the thickness changes.

In order to compare the desorption of the hydrogels with and without contact with cement paste blocks, hydrogels were also placed in the same drybox and the change in the mass of the hydrogels was monitored to obtain an estimate of water loss per initial hydrogel mass at the start of desorption. Assuming a small variation in the density of the hydrogels during desorption, at least in the initial stage of desorption, the desorption measurements based on volume change or mass change are comparable.

## Figures and Tables

**Table 1:** Compositions of the hydrogels used in the experiments.

	Monomers			Crosslinker	Initiator	
Hydrogel	Acrylic Acid (g)	Acrylamide (g)	Sodium hydroxide (g)	MBA (g)	Ammonium Persulfate (g)	Distilled Water (g)
H-1	9	1	1.215	0.025	0.064	50
H-2	5	5	0.675	0.025	0.064	50
H-3	1	9	0.135	0.025	0.064	50
